# Brain distribution of dipeptide repeat proteins in frontotemporal lobar degeneration and motor neurone disease associated with expansions in *C9ORF72*

**DOI:** 10.1186/2051-5960-2-70

**Published:** 2014-06-20

**Authors:** Yvonne S Davidson, Holly Barker, Andrew C Robinson, Jennifer C Thompson, Jenny Harris, Claire Troakes, Bradley Smith, Safa Al-Saraj, Chris Shaw, Sara Rollinson, Masami Masuda-Suzukake, Masato Hasegawa, Stuart Pickering-Brown, Julie S Snowden, David M Mann

**Affiliations:** Clinical and Cognitive Sciences Research Group, Institute of Brain, Behaviour and Mental Health, Faculty of Medical and Human Sciences, University of Manchester, Salford Royal Hospital, Salford, M6 8HD UK; Department of Neuropathology, Institute of Psychiatry, Denmark Hill, London, SE5 8AF UK; Department of Clinical Neuroscience, Institute of Psychiatry, Denmark Hill, London, SE5 8AF UK; Clinical and Cognitive Sciences Research Group, Institute of Brain, Behaviour and Mental Health, Faculty of Medical and Human Sciences, University of Manchester, A V Hill Building, Manchester, M13 9PT UK; Department of Neuropathology and Cell Biology, Tokyo Metropolitan Institute of Medical Science, 2-1-6 Kamikitazawa, Setagaya-ku, Tokyo, 156-8506 Japan

**Keywords:** Frontotemporal lobar degeneration, Motor neurone disease, C9ORF72, Dipeptide repeat proteins

## Abstract

A hexanucleotide (GGGGCC) expansion in *C9ORF72* gene is the most common genetic change seen in familial Frontotemporal Lobar Degeneration (FTLD) and familial Motor Neurone Disease (MND). Pathologically, expansion bearers show characteristic p62 positive, TDP-43 negative inclusion bodies within cerebellar and hippocampal neurons which also contain dipeptide repeat proteins (DPR) formed from sense and antisense RAN (repeat associated non ATG-initiated) translation of the expanded repeat region itself. ‘Inappropriate’ formation, and aggregation, of DPR might therefore confer neurotoxicity and influence clinical phenotype. Consequently, we compared the topographic brain distribution of DPR in 8 patients with Frontotemporal dementia (FTD), 6 with FTD + MND and 7 with MND alone (all 21 patients bearing expansions in *C9ORF72*) using a polyclonal antibody to poly-GA, and related this to the extent of TDP-43 pathology in key regions of cerebral cortex and hippocampus. There were no significant differences in either the pattern or severity of brain distribution of DPR between FTD, FTD + MND and MND groups, nor was there any relationship between the distribution of DPR and TDP-43 pathologies in expansion bearers. Likewise, there were no significant differences in the extent of TDP-43 pathology between FTLD patients bearing an expansion in *C9ORF72* and non-bearers of the expansion. There were no association between the extent of DPR pathology and *TMEM106B* or *APOE* genotypes. However, there was a negative correlation between the extent of DPR pathology and age at onset. Present findings therefore suggest that although the presence and topographic distribution of DPR may be of diagnostic relevance in patients bearing expansion in *C9ORF72* this has no bearing on the determination of clinical phenotype. Because TDP-43 pathologies are similar in bearers and non-bearers of the expansion, the expansion may act as a major genetic risk factor for FTLD and MND by rendering the brain highly vulnerable to those very same factors which generate FTLD and MND in sporadic disease.

**Electronic supplementary material:**

The online version of this article (doi:10.1186/2051-5960-2-70) contains supplementary material, which is available to authorized users.

## Introduction

Frontotemporal Lobar Degeneration (FTLD) is a clinical, pathological and genetically heterogeneous condition. The major clinical syndromes principally involve personality and behavioural change (behavioural variant frontotemporal dementia, or bvFTD) or language alterations of a fluent (semantic dementia) or non-fluent (progressive non-fluent aphasia) nature [1]. All three syndromes can be accompanied by Motor Neurone Disease (MND) though the combination of FTD and MND is most common [1]. Causative mutations have been identified in tau (*MAPT*) [2], progranulin (*GRN*) [3, 4], *CHMP2B* [5], and most recently in *C9ORF72* [6–8]. This latter genetic change is characterized by an expansion of a hexanucleotide (GGGGCC) repeat region in the first intron or the promoter region of *C9ORF72* gene, occurring in patients with either FTLD or MND, or a combination of both [6–8], and can number in excess of as many as 1500 repeats [9]. The expansion is found in about one in every twelve patients with FTLD and 1 in 10 patients with MND.

Pathologically, most FTLD cases with the expansion [6, 8, 10–13], like many non-mutational cases of FTLD [14, 15], show inclusion bodies within neurones (NCI) and glial cells of the cerebral cortex and hippocampus that contain the nuclear transcription factor, TDP-43. However, they also show a unique pathology within the hippocampus [10, 16, 17] and cerebellum [10–12, 16, 17] characterised by NCI that are TDP-43 negative, but immunoreactive to p62 protein. At least some of the target protein(s) within these p62-positive NCI are dipeptide repeat proteins (DPR) [17–20] formed from sense and antisense RAN (repeat associated non ATG-initiated) translation of the expanded repeat region itself [18–23]. ‘Inappropriate’ formation, and aggregation, of DPR may therefore confer neurotoxicity and influence clinical phenotype.

Previous studies on DPR in FTLD and MND have been largely limited to investigations on the hippocampus and cerebellum [17, 18], though one previous study [24] performed a wider topographic screen for DPR on 35 expansion cases with FTLD (n = 9) or MND (n = 8), or FTLD with MND (n = 18). This study failed to detect any significant differences in regional distribution or severity of DPR between each clinical group. In the present study, we too have determined how widely DPR are distributed throughout the brain in patients with FTLD and others with MND, and what cell types are affected, comparing the topographic distribution of DPR in patients with FTLD and MND in order to assess to what extent this distribution relates to the clinical expression of each disorder, and how it might relate to the underlying TDP-43 proteinopathy. Furthermore, because *TMEM106B* genotype has been claimed to be a genetic modifier of FTLD [25–29], and to protect *C9ORF72* carriers from FTD [25, 28, 29], we investigated whether this might influence both the distribution and severity of both DPR and TDP-43 pathologies in FTLD cases. We also performed a similar analysis in respect of Apolipoprotein E (*APOE*) genotype since there have many studies claiming an increased frequency of *APOE* ϵ4 allele in FTLD (see [30–33] for examples) and this might operate by facilitating pathological changes, as it does in Alzheimer’s disease where possession of *APOE* ϵ4 allele is associated with increased deposition of amyloid β protein [34] and cerebral amyloid angiopathy [35].

## Materials and methods

### Patients

Sixty seven patients were investigated in total. Fourteen patients with FTLD (cases#1-14, 9 males, 5 females), and 7 with MND (cases#41-47, 6 males, 1 female) bore expansions in *C9ORF72* (as evidenced by Southern blot and/or repeat primed PCR) (see Table 1 and Additional file 1: Table S1). We also investigated a further 14 other patients with FTLD (cases#27-40, 11 males, 3 females), and 20 other patients with MND (cases#48-67, 12 males, 8 females), all with no known mutation, and 12 patients with FTLD bearing a mutation in *GRN* (cases#15-26, 7 males, 5 females) (see Table 1 and Additional file 1: Table S1). All genetic analyses have been reported by us elsewhere [2, 3, 13, 36, 37]. Tissues from 39/40 FTLD and 23/27 of the MND cases were obtained from the Manchester Brain Bank through appropriate consenting procedures for the collection and use of the human brain tissues. All cases were from the North West of England and North Wales. The other FTLD case (case#14) and the further 4 MND cases (cases#44-47) were obtained from Institute of Psychiatry Brain Bank (London). Again, these were obtained through appropriate consenting procedures for the collection and use of the human brain tissues. The 40 patients with FTLD fulfilled Lund-Manchester clinical diagnostic criteria for FTLD [38, 39] and were consistent with recent consensus criteria [40, 41]. The 27 patients with MND fulfilled El Escorial criteria [42].

**Table 1:** **Mean (±SD) age at onset, death and duration of illness for groups of patients with FTD, FTD + MND or MND associated with expansions in** ***C9ORF72*** **, or mutations in** ***GRN*** **, or not known to be associated with any known FTLD or MND associated genes**

Group	M/F	Age at onset (y)	Age at Death (y)	Duration of illness (y)
*C9ORF72* FTD	7/1	61.1 ± 8.5	67.6 ± 7.0	6.5 ± 3.0
*C9ORF72* FTD + MND	2/4	58.8 ± 6.1	64.7 ± 5.8	5.8 ± 6.2
*C9ORF72* MND	6/1	55.8 ± 8.6	57.6 ± 7.6	2.8 ± 1.7
All *C9ORF72* cases	15/6	58.9 ± 7.8	63.4 ± 7.9	5.2 ± 4.1
*GRN* FTD	7/5	60.8 ± 5.9	69.7 ± 3.9	8.8 ± 3.8
Non-mutational FTD	2/2	60.8 ± 11.9	68.0 ± 15.6	7.3 ± 4.1
Non-mutational FTD + MND	9/1	59.3 ± 7.0	65.4 ± 8.2	6.2 ± 3.8
All non-mutational FTD	11/3	59.6 ± 12.7	63.4 ± 13.6	2.4 ± 1.1
Non-mutational MND	12/8	59.7 ± 8.2	66.1 ± 10.2	6.5 ± 3.8

FTD = Frontotemporal Dementia, FTD + MND + Frontotemporal dementia and Motor Neurone disease, MND = Motor Neurone Disease.

From clinical and neuropsychological assessments, 8 of the 14 FTLD cases with an expansion in *C9ORF72* had a pure/predominant bvFTD phenotype, whereas the other 6 showed a mixed bvFTD and MND phenotype. Five of the FTLD patients without known mutation showed bvFTD phenotype, 3 had a progressive non-fluent aphasia (PNFA) phenotype and 6 showed a mixed bvFTD and MND phenotype. Pathologically, among the *C9ORF72* expansion bearers with FTLD, all 8 patients with bvFTD had FTLD-TDP type A histology, whereas all 6 patients with FTD + MND had FTLD-TDP type B histology (according to Mackenzie et al. 2011 [43]). Of the 14 FTLD patients with no known mutation, 4 had FTLD-TDP type A histology (2 with bvFTD phenotype and 2 with PNFA), and 10 had FTLD-TDP type B histology (3 with bvFTD phenotype, 1 with PNFA phenotype and 6 with FTD + MND phenotype). All 12 patients bearing *GRN* mutation displayed FTLD-TDP type A histology; 6 had bvFTD phenotype and 6 had PNFA phenotype. All 27 patients with MND displayed characteristic TDP-43 pathology in mid brain and brainstem motor nerve nuclei, and in spinal cord (where this was available for study). These were usually skein-like in appearance, but occasionally more rounded, solid-appearing inclusions were also present.

### Histological methods

Paraffin sections were cut (at a thickness of 6 μm) from formalin fixed blocks of representative regions of brain to include (where available) frontal cortex (BA8/9), temporal cortex (BA21/22), cingulate gyrus, insular cortex, motor cortex, inferior parietal and occipital (BA17/18) cortex. Blocks were also cut from the amygdala and posterior hippocampus, basal ganglia (to include caudate nucleus, putamen, globus pallidus and thalamus), substantia nigra (to include oculomotor nucleus), pons (to include locus caeruleus and V cranial nerve nucleus), medulla (to include inferior olives and XII cranial nerve nucleus), cerebellum (with dentate nucleus) and cervical and lumbar spinal cord (where available).

Sections from each brain region were immunostained with a poly-GA antibody (courtesy of M Hasegawa), as described previously [44]. The antibody was used at dilution of 1:1000–1:3000. This antibody was raised against poly-(GA)_8_ peptide with cysteine at N-terminus, conjugated to *m*-maleimidobenzoyl-N-hydrosuccinimide ester-activated thyroglobulin. The thyroglobulin-peptide complex (200 μg) emulsified in Freund’s complete adjuvant was injected subcutaneously into a New Zealand White rabbit, followed by 4 weekly injections of peptide complex emulsified in Freund’s incomplete adjuvant, starting after 2 weeks after the first immunization. Immunoreactivity of the antisera was characterized by ELISA as follows. The peptide immunogens were coated onto microtiter plates. The plates were blocked with 10% fetal bovine serum (FBS) in PBS, incubated with the rabbit antisera diluted in 10% FBS/ PBS at room temperature for 1.5 h, followed by incubation with HRP-goat anti-rabbit IgG (Bio-Rad) at 1:3000 dilution, and reacted with the substrate, 0.4 mg/mL *o*-phenylenediamine, in citrate phosphate buffer (24 mM citric acid, 51 mM Na_2_HPO_4_). The absorbance at 490 nm was measured using Plate Chameleon (HIDEX). In addition, sections of temporal cortex with hippocampus from 11 (non-expansion bearing) cases with other histological and genetic forms of FTLD, other neurodegenerative disorders and healthy controls (see Table 1) were also immunostained for DRP with anti poly-GA antibody as ‘negative controls’. Antibodies were employed in standard IHC protocol, though antigen unmasking was performed by pressure cooking in citrate buffer (pH 6, 10 mM) for 30 minutes, reaching 120 degrees Celsius and >15 kPa pressure.

Further sections of frontal cortex and temporal cortex with hippocampus were immunostained for both phosphorylated (at Ser 409/410), and non-phosphorylated, TDP-43 (rabbit polyclonal antibodies (pS409/410-2 antibody, Cosmo Biotech Ltd, Tokyo, Japan and 10782-2-AP antibody, Proteintech, Manchester, UK, respectively – at 1:3000 and 1:1000, respectively).

### Pathological assessment

The presence of DPR immunostained NCI within nerve cells was assessed at ×20 magnification in those brain regions where all cases could be represented, according to:

- 0 = no DPR immunostained NCI present in any field.
- 0.5 = rare/single DPR immunostained NCI present in entire section.
- 1 = a few DPR immunostained NCI present, in some but not all fields.
- 2 = a moderate number of DPR immunostained NCI present in each field.
- 3 = many DPR immunostained NCI present affecting many cells in each field.
- 4 = very many DPR immunostained NCI present, affecting nearly all cells in every field.

Scores per assessed area were summated across those regions where these were available for all 21 individuals with *C9ORF72* expansions. Brain regions were grouped on an anatomical or a ‘functional’ basis. Hence, scores from frontal, temporal, cingulate, insular, parietal and occipital cortical regions were summated to generate a total ‘cortical’ score for each case. Scores from hippocampus and adjacent regions of subiculum, entorhinal cortex and fusiform gyrus were summated to give a total medial temporal lobe score for each case. Scores for caudate nucleus, putamen, globus pallidus, thalamus, substantia nigra, locus caeruleus and dorsal raphe were summated to give a total ‘subcortical’ score. Scores in motor cortex and in X and XII cranial nerve nuclei were summated to give a total ‘motor’ score. Scores in cerebellar granule and Purkinje cells, and in cells of the dentate nucleus, inferior olives and pontine nuclei were summated to give a total ‘cerebellar’ score. Due to the unavailability of tissue in all cases, it was not possible to include scores for amygdala (absent from 5/21 cases) or spinal cord (absent from 10/21 cases) within the medial temporal lobe or motor region analyses, respectively.

The frequency of TDP-43 pathological changes (as NCI and neurites, where present) in each of frontal and temporal cortex (pyramidal cells of layers II) and hippocampus (dentate gyrus granule cells), was scored semi-quantitatively according to:

- 0 = no TDP-43 immunostained NCI and/or neurites.
- 0.5 = rare/single TDP-43 immunostained NCI and/or neurite present in entire section.
- 1 = very few TDP-43 immunostained NCI and/or neurites.
- 2 = a moderate number of TDP-43 immunostained NCI and/or neurites.
- 3 = many TDP-43 immunostained NCI and/or neurites, affecting many cells in every field.
- 4 = very many immunostained NCI and/or neurites present, affecting nearly all cells in every field.

TDP-43 pathology cores per assessed area were summated across those regions where these were available for all individuals. Hence, scores from dentate gyrus of hippocampus, and from frontal and temporal cortex, were summated (in all except 4 cases where dentate gyrus was not available) to give a total TDP-43 pathology score for each case.

### Genetic analysis

DNA was extracted from blood or frozen brain tissue by routine phenol-chloroform extraction. The *TMEM106B* assay was genotyped by allelic discrimination using the Applied Biosystems pre-developed assay cat number C_7604953_10. Genotyping was carried out using the Applied Biosystems 7900, and genotypes were assigned automatically using the SDS 2.3 software. *APOE* was genotyped according to Wenham [45].

### Statistical analysis

Rating data was entered into an excel spreadsheet and analyzed using Statistical Package for Social Sciences (SPSS) software (version 17.0). Kruskal-Wallis or Mann–Whitney test was used to compare inclusion scores between several groups or pairs of groups, respectively. All correlations were performed using Spearman rank correlation test. In all instances, a p-value of less than 0.05 was considered statistically significant.

## Results

### Demographics

Mean ages at onset, death and duration of illness for *C9ORF72* associated FTD, FTD + MND and MND groups, FTD associated with *GRN* mutation, non-mutational FTD, FTD + MND and MND groups are shown in Table 1.

ANOVA comparison of age at onset, death and duration of illness between all 7 diagnostic groups revealed no significant differences in age at onset (F_6,51_ = 0.27, p = 0.946) or death (F_6,56_ = 1.4, p = 0.227), though duration of illness differed between the groups (F_6,51_ = 4.3, p = 0.001). As would be expected, post-hoc analysis showed that duration of illness was significantly less in *C9ORF72* cases with MND than *C9ORF72* associated FTD (p = 0.014), but not less than *C9ORF72* associated FTD + MND (p = 0.281), nor was there any difference in disease duration between *C9ORF72* associated FTD and *C9ORF72* associated FTD + MND (p = 0.815). Again, as expected, non-mutational MND showed a shorter duration of illness than non-mutational FTD (p = 0.022) and non-mutational FTD + MND (p = 0.013). However, there were no significant differences in duration of illness between *C9ORF72* associated FTD, non-mutational FTD or *GRN* associated FTD (F_2,37_ = 1.6, p = 0.215), or between *C9ORF72* associated FTD + MND and non-mutational FTD + MND (p = 0.900), or between *C9ORF72* associated MND and non-mutational MND (p = 0.605).

ANOVA comparison of age at onset, death and duration of illness between *C9ORF72* associated FTD, FTD + MND and MND groups alone revealed no significant group differences in age at onset (F_2,17_ = 0.77, p = 0.477) or duration of illness (F_2,17_ = 1.4, p = 0.227), though duration of illness tended to differ between the 3 groups (F_2,18_ = 4.1, p = 0.045).

### Cytological observations

All cases had been previously classified on the basis of the type and distribution of TDP-43 immunoreactive changes according to Mackenzie et al. 2011 [43], and hence showed TDP-43 histological changes typical of the group in which they had been placed. These have been well described previously, both by ourselves and others, and are therefore not further detailed in the present study.

**Figure 1:**
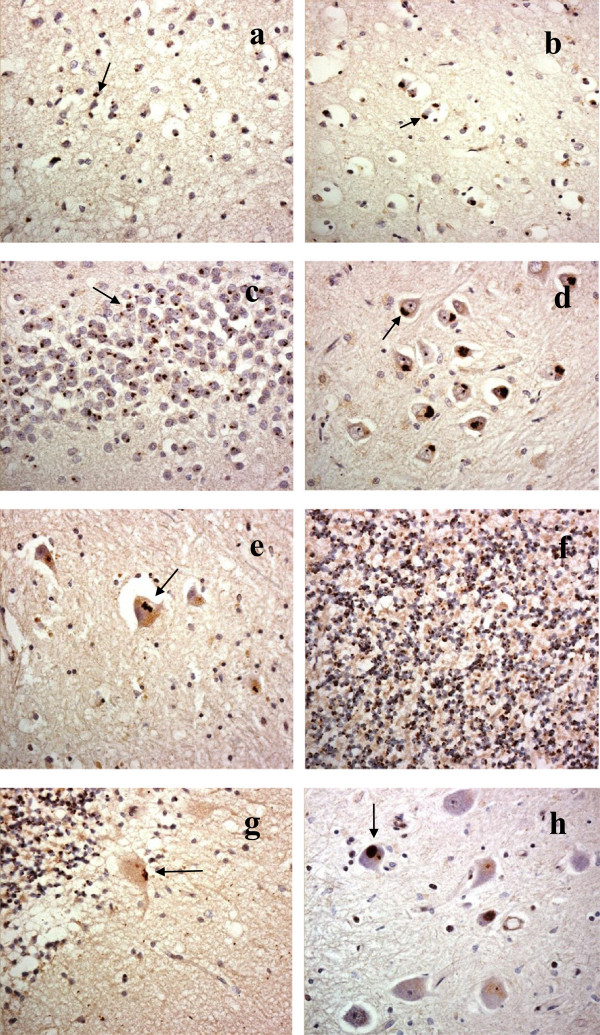
**Topographic brain distribution of dipeptide repeat proteins (poly-GA) in patient #9 with Frontotemporal dementia associated with an expansion in** ***C9ORF72.*** Regions shown are frontal cortex layer II **(a)**, frontal cortex layer V **(b)**, dentate gyrus **(c)** and area CA4 **(d)** of hippocampus, ventrolateral nucleus of thalamus **(e)**, granule cells **(f)** and Purkinje cells **(g)** of cerebellum, dentate nucleus **(h)**. Immunoperoxidase-haematoxylin ×40 microscope magnification.

DPR were characteristically present in all FTLD and MND cases previously known to bear expansions in *C9ORF72*, but none were seen in any of the cases bearing *GRN* mutations, nor in any of the other FTLD cases or MND cases not known to be associated with any FTLD or MND linked mutation.In cases of FTD and FTD + MND, DPR were observed to be most frequent within the cerebral neocortex, hippocampus and cerebellum. They were infrequent or absent in basal ganglia regions, and were rare or usually absent in mid brain, brainstem, medulla and spinal cord regions. DPR were present in neuronal cytoplasmic inclusions (NCI) throughout all cortical layers in all regions of the cerebral cortex examined. In outer cortical layers they were mostly present in small non-pyramidal neurons appearing as dots or clusters of granules (Figure 1a), whereas in the deeper cortical layers DPR were again present in smaller non-pyramidal neurons as clusters of granules, but in the larger pyramidal cells they often adopted a more star-shaped or spicular appearance (Figure 1b). Granular type DPR were particular numerous in parietal and occipital cortex and in motor cortical regions in most cases, and common in frontal and temporal cortex when these areas were not severely degenerated, though in cases where these regions were badly degenerated DPR were much less frequent.In the hippocampus, DPR were present as abundant, small, rounded NCI within granule cells of the dentate gyrus (Figure 1c), though more spicular or granular inclusions were commonly seen within pyramidal cells of areas CA4 and CA2/3 (Figure 1d), becoming less frequent in CA1 region and subiculum. Again neurons containing small clusters of DPR were present throughout all layers of the entorhinal cortex. Small granular or spicular NCI were widespread within the amygdala, though usually only to a moderate extent, and less severely than in the hippocampus.Generally, DPR were sparse within basal ganglia regions. They were occasionally present as spicular NCI in small neurons in the caudate nucleus and putamen, and in larger neurons of the globus pallidus but were more common in the thalamus, especially those of the ventrolateral nuclei (Figure 1e). DPR were rarely (if ever) seen in cells of the substantia nigra, locus caeruleus, nucleus basalis of Meynert, dorsal raphe nucleus, basalis pontis, in motor nuclei of III, IV, V, X or XII cranial nerves, or in anterior horn cells of the spinal cord (where available for study). However, in a few cases DPR were occasionally seen as spicular NCI in neurons of the inferior olives.

**Figure 2:**
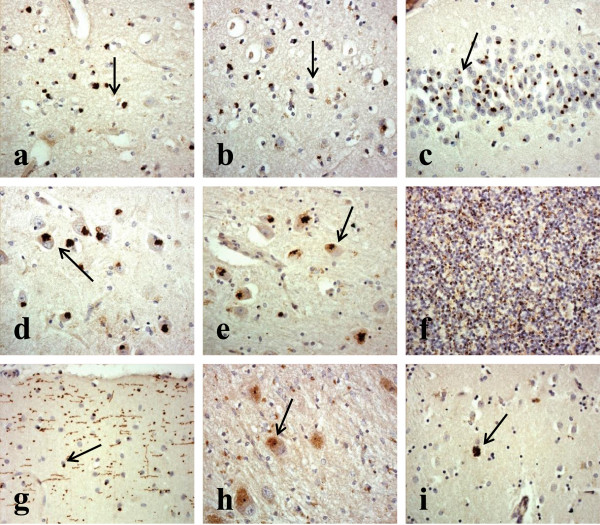
**Topographic brain distribution of dipeptide repeat proteins (poly-GA) in patient #23 with Motor Neurone Disease associated with an expansion in** ***C9ORF72.*** Regions shown are frontal cortex layer II **(a)**, frontal cortex layer V **(b)**, dentate gyrus **(c)** and area CA4 **(d)** of hippocampus, ventrolateral nucleus of thalamus **(e)**, granule cells **(f)** and Purkinje cells **(g)** of cerebellum, dentate nucleus **(h)** and putamen **(i)**. Immunoperoxidase-haematoxylin ×40 microscope magnification.

In the cerebellum, DPR were widespread within granule cells of the cerebellum (Figure 1f). As with the cerebral cortex, more granular looking NCI were usually present in basket cells, with occasional spicular NCI being seen in Purkinje cells (Figure 1g) and neurones in the dentate nucleus (Figure 1h), but none were seen within Golgi neurones, or within Bergmann glia. A punctate, or filamentous, staining was also seen within the molecular layer of the cerebellum, this probably relating to parallel projection fibres (Figure 1g). A similar distribution of DPR was observed in the 7 MND cases bearing expansions in *C9ORF72* to that seen in the FTD and FTD + MND cases (Figure 2).

### Comparisons of DPR ratings

Composite rating scores for DPR pathology are shown for each case in Table 2. When Comparisons of rating scores for DPR, showed no significant differences in total severity scores for DPR between FTD, FTD + MND and MND cases for cortical (χ^2^ = 0.19, p = 0.911), hippocampal (χ^2^ = 3.7, p = 0.160), motor (χ^2^ = 2.3, p = 0.323), subcortical (χ^2^ = 2.56, p = 0.279) or cerebellar (χ^2^ = 2.3, p = 0.318) regions, or for total score summated across all 5 subregions (χ^2^ = 1.4, p = 0.487) (Table 2). Similarly, when analysed by region, there were no significant differences in DPR scores between any of the cortical regions investigated, either over all cases, or according to clinical subgroups.

**Table 2:** **Summated scores for DPR pathology ratings in Cortical, Medial Temporal, Motor, Subcortical and Cerebellar regions, as well as total score across all 5 regions, for patients with FTD (cases #1-8), FTD + MND (cases#9-14) and MND (cases#41-47) associated with expansion in** ***C9ORF72***

Case ID#		Summated DPR score					
	Pathology	Cortex	Medial temporal	Motor	Subcortical	Cerebellar	Total
1	FTLD-TDP A	16	12	3	3	5	39
2	FTLD-TDP A	12	14	3	1	5.5	35.5
3	FTLD-TDP A	15	18	3	5	7.5	48.5
4	FTLD-TDP A	13	12	2	2.5	3.5	33
5	FTLD-TDP A	12	11	1	3	3	30
6	FTLD-TDP A	11	9	1.5	3	2	26.5
7	FTLD-TDP A	12	9.5	1	5.5	3.5	31.5
8	FTLD-TDP A	22	23	2	4	5.5	55.5
9	FTLD-TDP B	20	15.5	2	1	4.5	43
10	FTLD-TDP B	12	11	2	2	4	31
11	FTLD-TDP B	11	10.5	2	1.5	4	29
12	FTLD-TDP B	18	16	3.5	4.5	5	47
13	FTLD-TDP B	11	11	2	3.5	2	29.5
14	FTLD-TDP B	16	15	3	4.5	6.5	45
41	MND	16	13	3	6	5.5	43.5
42	MND	15	13	3	3	1	35
43	MND	14	18	3	4	3	42
44	MND	12	18	2.5	5	3	40.5
45	MND	13	12	3	1.5	4.5	34
46	MND	11	19	2	4.5	3	39.5
47	MND	23	23	3	5	3.5	57.5

When all cases were grouped, there were significant correlations between the severity of DPR pathology and age at onset of disease for cortical (r_s_ = −0.620, p = 0.004), hippocampal (r_s_ = −0.537, p = 0.015), motor (r_s_ = −0.482, p = 0.031) and for total score summated across all 5 subregions (r_s_ = −0.583, p = 0.016), but not for subcortical (r_s_ = −0.218, p = 0.356) or cerebellar (r_s_ = −0.137, p = 0.318) regions. However, there was no significant correlation between the severity of DPR pathology and duration of disease, except weakly so in cerebellum (r_s_ = −0.493, p = 0.027).

Comparisons of DPR severity scores according to possession of at least one copy of *APOE* ϵ4 allele were performed on 12/14 FTLD cases and 3/7 MND cases (for whom DNA was available for genotyping). No significant differences in DPR severity scores were found between ϵ4 allele bearers and non-bearers, either overall (p = 0.240) or according to subregion (cortical (p = 0.130), hippocampal (p = 0.189), motor (p = 0.887), subcortical (p = 0.506) or cerebellar (p = 0.640).

Due to availability of DNA, *TMEM106B* genotypes were only available for 7 of the 14 FTLD cases, and 1 of the 7 MND cases, bearing *C9ORF72* expansions, 9 of the 12 *GRN* mutation carriers, and 10 of the 14 FTLD cases and 1 of the 20 MND cases without known mutation. Because *TMEM106B* SNPs rs1020004, rs1990622 and rs6966915 were in complete linkage disequilibrium, pathological analyses were confined to rs 1990622. Unfortunately, only one of the FTLD, and none of the MND cases, irrespective of type or presence of mutation, was homozygous for the minor allele at any SNP. Of the *C9ORF72* expansion bearers, 4 of the FTLD cases were homozygotes for the major allele and 3 were heterozygotes, with the MND case being homozygous for the major allele. Of the *GRN* mutation bearers, 5 of the FTLD cases were homozygotes for the major allele and 4 were heterozygotes. Of the non-mutational FTLD cases, 6 were homozygotes for the major allele, 2 were heterozygotes and 1 was homozygous for the minor allele.

Nonetheless, comparisons of DPR severity scores were performed between FTLD cases heterozygous (TC) and homozygous (TT) for the major allele. No significant differences in DPR severity scores were found between bearers of TC or TT genotypes, either overall (p = 0.881) or according to subregion (cortical (p = 0.881), hippocampal (p = 0.549), motor (p = 0.495), subcortical (p = 0.546) or cerebellar (p = 0.878).

### Comparisons of TDP-43 ratings

Composite rating scores for TDP-43 pathology are shown for each case in Table 3. There were no significant differences in the severity of TDP-43 pathology scores between FTLD cases bearing expansions in *C9ORF72*, FTLD cases without expansions, and FTLD cases with *GRN* mutation for either frontal cortex (χ^2^ = 1.95, p = 0.377), temporal cortex (χ^2^ = 1.94, p = 0.379) or dentate gyrus of hippocampus (χ^2^ = 1.55, p = 0.461), or as total TDP-43 severity score (χ^2^ = 1.82, p = 0.403). Moreover, there were no significant differences in the severity of TDP-43 pathology scores between FTLD cases with type A histology bearing an expansion in *C9ORF72*, FTLD cases with type A histology but without expansion in *C9ORF72* mutation, and FTLD cases with *GRN* mutation and type A histology for either frontal cortex (χ^2^ = 2.27, p = 0.321), temporal cortex (χ^2^ = 0.66, p = 0.719) or dentate gyrus of hippocampus (χ^2^ = 5.45, p = 0.065), or as total TDP-43 severity score (χ^2^ = 2.19, p = 0.334). Similarly, there were no significant differences in the severity of TDP-43 pathology scores between FTLD cases with type B histology bearing an expansion in *C9ORF72* and FTLD cases with type B histology but without expansion in *C9ORF72* mutation for either frontal cortex (p = 0.570), temporal cortex (p = 0.147) or dentate gyrus of hippocampus (p = 0.344), or as total TDP-43 severity score (p = 0.254).

**Table 3:** **Summated scores for TDP-43 pathology ratings in Frontal and Temporal cortex, and Dentate Gyrus, as well as total score across all 3 regions, for patients with FTD (cases #1-8) or FTD + MND (cases#9-13) associated with** ***C9ORF72*** **expansions, and for patients with FTLD due to** ***GRN*** **mutation (case#15-26) or not associated with any known mutation (cases#27-40)**

Case ID#	Gene	Pathology	TDP-43 score			
			DG	TCX	FCX	Total
1	*C9ORF72*	FTLD-TDP A	1	4	3	8
2	*C9ORF72*	FTLD-TDP A	2	4	3	8
3	*C9ORF72*	FTLD-TDP A	2	1	3	6
4	*C9ORF72*	FTLD-TDP A	1	3	3	7
5	*C9ORF72*	FTLD-TDP A	1	1	1	3
6	*C9ORF72*	FTLD-TDP A	2	2	2	6
7	*C9ORF72*	FTLD-TDP A	1	1	2	4
8	*C9ORF72*	FTLD-TDP A	2	2	3	7
9	*C9ORF72*	FTLD-TDP B	3	2	2	7
10	*C9ORF72*	FTLD-TDP B	4	4	4	12
11	*C9ORF72*	FTLD-TDP B	4	3	3	10
12	*C9ORF72*	FTLD-TDP B	1	2	2	5
13	*C9ORF72*	FTLD-TDP B	na	3	3	na
15	*GRN*	FTLD-TDP A	na	4	4	na
16	*GRN*	FTLD-TDP A	na	2	2	na
17	*GRN*	FTLD-TDP A	4	3	3	10
18	*GRN*	FTLD-TDP A	2	2	2	6
19	*GRN*	FTLD-TDP A	3	3	3	9
20	*GRN*	FTLD-TDP A	1	4	4	9
21	*GRN*	FTLD-TDP A	2	4	4	10
22	*GRN*	FTLD-TDP A	4	2	1	7
23	*GRN*	FTLD-TDP A	2	1	3	6
24	*GRN*	FTLD-TDP A	2	0	1	3
25	*GRN*	FTLD-TDP A	3	4	4	11
26	*GRN*	FTLD-TDP A	na	3	4	na
27	No mutation	FTLD-TDP A	2	3	1	6
28	No mutation	FTLD-TDP A	3	4	4	11
29	No mutation	FTLD-TDP A	2	2	2	6
30	No mutation	FTLD-TDP A	1	1	1	3
31	No mutation	FTLD-TDP B	4	4	4	12
32	No mutation	FTLD-TDP B	0	3	4	7
33	No mutation	FTLD-TDP B	3	0	1	4
34	No mutation	FTLD-TDP B	2	1	1	4
35	No mutation	FTLD-TDP B	3	2	4	9
36	No mutation	FTLD-TDP B	4	1	1	6
37	No mutation	FTLD-TDP B	2	2	2	6
38	No mutation	FTLD-TDP B	1	0	1	2
39	No mutation	FTLD-TDP B	1	2	3	6
40	No mutation	FTLD-TDP B	3	3	3	9

Within the *C9ORF72* expansion carriers, there were no significant correlations between the severity of TDP-43 pathology and either age at onset of disease, or duration of illness, for either cortical, hippocampal or total scores. Neither was there any correlation between TDP-43 scores and clinical phenotype (ie FTD versus FTD + MND).

Comparisons of TDP-43 severity scores according to possession of at least one copy of *APOE* ϵ4 allele were performed on 36/39 FTLD cases (for whom DNA was available for genotyping), irrespective of the presence or absence of mutation. No significant differences in the severity of TDP-43 pathology scores were seen between FTLD cases with *APOE* ϵ4 allele and FTLD cases without *APOE* ϵ4 allele for either frontal cortex (p = 0.187), temporal cortex (p = 0.293) or dentate gyrus of hippocampus (p = 0.171), or as total TDP-43 severity score (p = 0.261). Similarly, comparisons of TDP-43 severity scores according to heterozygosity (TC) or homozygosity (TT) for major allele in *TMEM106B* were performed on 24/39 FTLD cases (where these data were available), irrespective of the presence or absence of mutation. No significant differences in the severity of TDP-43 pathology scores between FTLD cases with TC and TT genotypes were seen for either frontal cortex (p = 0.301), temporal cortex (p = 0.592) or dentate gyrus of hippocampus (p = 0.768), or as total TDP-43 severity score (p = 0.519).

## Discussion

There are two major outcomes from this study. Firstly, that in cases of FTLD or MND bearing expansions in *C9ORF72*, neither the topographical distribution, nor the relative severity, of DPR differs between cases of FTD alone, FTD + MND or MND alone. Secondly, neither the morphological appearance, nor relative severity, of TDP-43 immunoreactive changes differ in cases of FTLD bearing an expansion in *C9ORF72* from that in non-expansion bearing cases of FTLD (ie sporadic forms of FTLD), or from cases of FTLD bearing mutations in *GRN*. These findings support the observations of Mackenzie and colleagues [24] who likewise noted no differences in either DPR or TDP-43 pathology between expansion bearing cases of FTD alone, FTD + MND or MND alone. Such observations call into question the relevance of DPR pathology in determining clinical phenotype, and the role of the expansion *per se* in causing FTLD or MND.

Expansions in *C9ORF72* have been postulated to invoke disease by 3, mutually non-exclusive, mechanisms: firstly involving a loss of C9orf72 protein through haploinsufficiency [6], secondly by RNA toxicity through sequestration of RNA species by the expanded sequences [6, 21], and thirdly through cytotoxicity from formation and accumulation of DPR [18–20, 22, 23]. While this latter suggestion is attractive, and has parallels with other aggregating brain proteins such as tau, TDP-43, α-synuclein, there is strong evidence that argues against such an effect.

Firstly, in both the present, and in other studies [24], the distribution of DPR, as evidenced by poly-GA immunostaining, does not parallel that of neurodegeneration (ie the TDP-43 proteinopathy) in either FTLD or MND. Indeed, in both disorders the greatest severity of DPR lies in brain regions such as the granule cell layer of the cerebellum, the hippocampal dentate gyrus and CA4 pyramidal cells, and the parietal and occipital cortex, with similar observations being recorded for poly-GP and poly-GR antibodies [17]. Such affected regions of the brain have hitherto not been considered to be involved in the (TDP-43) pathological process, and are brain regions which do not show obvious clinical repercussions, which might be anticipated if the DPR were principal in driving a neurodegenerative process, although there have been reports of cerebellar atrophy in expansion bearers [46, 47].

Secondly, it is hard to conceive how a seemingly identical topographical pattern of DPR pathology could determine, or even predispose towards, such diverse clinical phenotypes as FTD or MND. To explain such an apparent paradox, Mackenzie et al. 2013 [24] invoked the argument that the visible DPR protein aggregates conferred a neuroprotective effect in functionally preserved brain regions with high DPR loads, such as cerebellum, hippocampus and occipital cortex, through sequestration of soluble toxic species. In the present study, we were unable to show any significant differences in DPR load between any of the neocortical regions investigated, these being as equally high in functionally disturbed areas such as frontal, temporal and motor cortex, as those in apparently functionally normal regions such as parietal and occipital cortex. It is therefore difficult to reconcile such observations with any putative neuroprotective effect, since if this were indeed the case then it might be anticipated that visible DPR should be lower in cortical regions, such as frontal and temporal cortex, showing functional change and neurodegeneration, in which any putative neurotoxic effects of soluble (oligomeric) precursors had not been so restricted. Nonetheless, there does appear to be some clinical distinctions between expansion bearers and non-bearers in both FTLD and MND, in as much as expansion bearers in both conditions are more likely to display psychosis [13, 48, 49]. Concerning the present cases, there was no difference between the level of DPR pathology in those cases presenting with a florid psychosis compared to those where psychosis was not apparent (data not shown), suggesting that the presence and/or extent of DPR pathology is neither a determinant, nor a modulator, of psychosis.

The second observation from this study indicates that as far as the TDP-43 proteinopathy is concerned, there are again no distinctions in pathology between expansion bearers and non-bearers of the expansion, either overall or more specifically in respect of FTLD-TDP type A or type B histologies. This also supports observations by Mackenzie et al. [24] and further calls into question the role of the expansion *per se*. Although, there is no *a priori* reason to suspect that patients with an expansion in *C9ORF72* should ‘naturally’ carry a higher burden of TDP-43 pathology than non-expansion carriers, it is nonetheless important to observe that such differences do indeed not exist. Moreover, if the expansion were directly driving a TDP-43 proteinopathy, it is hard to reconcile this with observations of different TDP-43 histologies. FTLD-TDP Type B (involving a preponderance of neuronal cytoplasmic inclusions) is the most common TDP-43 histological subtype associated with the expansion [12, 24], though a significant number of cases display type A histology [17], and rare cases with type C histology have been described [12]. How such diverse histological patterns could stem from a common genetic root is puzzling.

An alternative explanation of the role of *C9ORF72* could be that the expansion acts as a risk factor for FTLD and MND, though does not drive the (TDP-43) pathological process directly, acting more as a ‘gatekeeper’ to disease, rendering the brain susceptible to the ‘development’ of all sporadic forms of FTLD-TDP, and sporadic forms of MND associated with TDP-43. Such a scenario could accommodate the present observations of the different histological types of TDP-43 proteinopathy being associated with disease, and the relative proportions with which they occur, and with findings that the extent of TDP-43 pathology is the same in expansion and non-expansion bearers both in terms of either type A or type B histologies.

There is other evidence in support of this latter line of argument. Firstly, although rare, pathologies other than FTLD or MND, such as corticobasal degeneration [13, 17], Alzheimer’s disease ([50–52], but see [44]), Parkinson’s disease [53] and Huntington disease-like conditions [9], have been associated with expansions in *C9ORF72*. Indeed, there has been one Belgian case with *C9ORF72* expansion and clinical FTLD which lacks detectable TDP-43 pathology, but shows FTLD-UPS pathology [7]. Secondly, there appears to be no link between expansion size and TDP-43 histological type [17], or clinical phenotype [9]. Thirdly, there appears to be a higher than expected coincidence of repeat expansions in individuals carrying other genetic variants involving mutations in *GRN* with *C9ORF72* [54, 55] or *MAPT* with *C9ORF72* [54–57] (so called oligogenic inheritance), suggesting that another ‘hit’ may be necessary for clinical disease, yet in these dual mutation cases, apart from the DPR changes, either a TDP-43 proteinopathy, or tauopathy, typical of the accompanying (*GRN* or *MAPT*) mutation prevails.

The correlation between age at onset and severity of DPR pathology, unrelated to disease duration, is interesting though the reason for this is unclear. It is possible that there is a more severe ‘expression’ of the expansion in younger individuals leading to a greater level of production and accumulation of DPR, or that the dipeptides aggregate more efficiently into NCI perhaps due to variations in length of DPR monomers formed as a result of differences in translation efficiency with age during RAN translation.

*TMEM106B* genotype (ie homozygosity for the minor allele) has been claimed to be a genetic modifier of FTLD in both *GRN* and *C9ORF72* mutation carriers [25, 26, 28, 29], and to protect *C9ORF72* carriers from FTD [25, 28, 29]. Indeed, van Blitterwijk et al. [28] reported that homozygous carriers of the minor protective *TMEM106B* allele with FTLD-TDP type A histology appeared to have a lower TDP-43 burden than homozygous carriers of the major allele. Therefore, we investigated whether variations at this locus might influence both the distribution and severity of both DPR and TDP-43 pathologies in FTLD cases associated with either *C9ORF72* expansions or *GRN* mutations. Unfortunately, no homozygous bearers of *TMEM106B* minor allele were available in the present study for either mutation, but we did not find any differences in DPR and TDP-43 pathology between heterozygotes and those homozygous for the major allele suggesting that heterozygosity, at least, does not confer a ‘partial’ protection in either *C9ORF72* or *GRN* mutation carriers. Similarly, although possession of at least one copy of *APOE* ϵ4 allele has been (variably) claimed to increase risk of FTLD [30–33], we found no significant differences in extent of either DPR or TDP proteinopathy between ϵ4 allele bearers and non-bearers, implying that any putative risk associated with ϵ4 allele does not operate through facilitating the pathological changes of FTLD.

How the expansion might act in a ‘gatekeeper’ role is not clear. Much attention has been levied towards a determination of the nature and effect of DPR *in vivo*, and *in vitro*, as well as the role RNA foci might play in causing the disease. However, there is evidence of C9orf72 haploinsufficiency in expansion carriers [6], though given the paucity of knowledge surrounding the role and function of C9orf72, it is difficult to ascribe precise meaning to such a putative loss of protein. A haploinsufficiency state might impair vital ‘protective’ brain functions. Alternatively, the formation of toxic RNA foci could likewise render cells vulnerable [6, 21, 23, 58], though against this are observations that such a process, like DPR formation, is disseminated widely throughout the brain, and not solely confined to those neuronal populations vulnerable to TDP-43 proteinopathy. Present knowledge suggests C9orf72 belongs to the differentially expressed in normal and neoplastic (DENN)-like family of proteins, these being GDP/GTP exchange factors which lead to activation of Rab-GTPases and maintenance of vesicular trafficking [59]. In this regard, it is interesting that the most recent GWAS for FTLD has highlighted variations in *RAB38* as a risk factor for bvFTD [60]. RAB38 has been suggested to mediate protein trafficking to lysosomal-related organelles [61, 62] and maturation of phagosomes [63], and impairment in these processes might elicit cargo accumulation in early endosomes, with downstream effects on recycling/degradative pathways [61]. Indeed, an association with lysosomal processes in FTLD has previously been suggested by two studies on *GRN* [64] and *TMEM106B* [65]. Considering that endolysosomal homeostasis is essential for the health of neurons, functional links between RAB38, TMEM106B, PGRN and FTLD imply disturbances in C9orf72 may trigger or promote autophagosomal/lysosomal dysfunctions, thereby playing a key role in the onset and/or progression of the disease.

Nonetheless, if such a ‘gatekeeper role’ is true, then the almost complete penetrance of the mutation needs to be explained. In this respect the expansion might be viewed as providing an ‘open door’ to disease, but without actually causing disease directly, and in this regard the DPR pathology may be a ‘red herring’ without pathogenetic consequence. Clearly, until we know more about the normal function of C9orf72 protein, what cells it is present in, and where in the cell it is located, all is mere speculation. The concept of the expansion being a risk factor for disease, rather than a cause, is tempting since it would neatly accommodate the observed diversity of clinical and histological subtypes associated with the expansion. However, were the expansion to act as a risk factor, it appears to act (almost) selectively for FTLD and MND since expansions are only infrequently, and then maybe only coincidentally, seen in more common disorders such as Alzheimer’s and Parkinson’s disease [50–53]. Therefore, while it is clear that expansions in *C9ORF72* are not without ‘pathological expression’, what DPR might translate into in clinical terms is not clear, and whether these structures confer anything beyond diagnostic utility still remains to be demonstrated.

## Electronic supplementary material

Additional file 1: **Selected clinical, pathological and genetic details for 40 cases of Frontotemporal Lobar degeneration and 27 cases of Motor Neurone disease.** (XLSX 18 KB)
